# Measuring long-range contacts in a fully protonated protein at 105 kHz magic angle spinning

**DOI:** 10.1007/s10858-025-00477-8

**Published:** 2025-09-26

**Authors:** Zainab O. Mustapha, Eren H. Ozturk, Benjamin E. Lefkin, Diana Grajeda, Andrew J. Nieuwkoop

**Affiliations:** Department of Chemistry and Chemical Biology, Rutgers, The State University of New Jersey, Piscataway, NJ 08854 USA

**Keywords:** ^1^H Detection, Solid-state NMR, Fully protonated systems, Very fast MAS, Long-range contacts

## Abstract

The use of ^1^H detection, made possible by very fast magic-angle spinning (MAS), has revolutionized the field of biomolecular solid-state NMR. In the past, ^1^H detection was often paired with deuteration schemes to achieve the highest possible resolution needed for protein structural characterization. However, with modern probes capable of MAS rates over 100 kHz, deuteration is no longer required, resulting in a need to measure long-range distances in fully protonated systems. In this study, we evaluate the potential of two 3D pulse sequences, (H)NCOH and (H)NCAH, to measure long-range C-H correlations in a fully protonated protein sample at a MAS rate of 105 kHz. Our results show that the (H)NCOH spectrum contains multiple sequential and structurally relevant long-range CO–H contacts for each residue, capturing H^N^ contacts up to 6 Å despite transfers to side chain protons. Conversely, the (H)NCAH spectrum yields fewer Cα-H^N^ correlations, with those present mostly from intraresidue aliphatic proton contacts. Therefore, in protonated proteins, the extensive ^1^H network leads to dipolar truncation in the Cα-H experiment, while the CO–H correlations observed are comparable to those in deuterated samples. These findings highlight the feasibility of conducting distance measurements based on long-range cross polarization, on more accessible and affordable samples, expanding the scope of proton detection for systems where deuteration and back-exchange are not possible.

**Supplementary Information:**

The online version contains supplementary material available at 10.1007/s10858-025-00477-8.

## Introduction

Magic angle spinning solid-state NMR (MAS ssNMR) continues to be a powerful tool for determining the structure of certain classes of proteins which have proved challenging for other experimental techniques to investigate. The adoption of ^1^H detection, made possible by higher MAS rates, has further enabled the study of larger protein systems. When combined with deuteration schemes, moderate MAS frequencies can reduce proton linewidths to around 20 Hz, making structural characterization feasible (Zhou et al. 2007; Linser et al. 2008). However, deuteration and back-exchange are challenging or even impossible in some systems, particularly in membrane proteins with regions of restricted solvent accessibility (Barbet-Massin et al. 2014). Moreover, deuteration is costly, requires complex expression methods, and limits the detection of side chains (Andreas et al. 2016a). Very fast MAS spinning offers an alternative to substantial proton reduction in samples and serves as an effective tool for proton homonuclear decoupling, addressing the significant challenges posed by strong proton-proton dipolar couplings to spectral resolution (Reif 2012; Xue et al. 2017; Linser et al. 2011).

With an increase in spinning rates from 60 kHz to over 100 kHz, it has become possible to complete the sequential backbone chemical shift assignments using a fully protonated sample due to the enhanced resolution and sensitivity of proton-detected experiments (Andreas et al. 2016a; Andreas et al. 2016b; Agarwal et al. 2014; Penzel et al. 2015; Penzel et al. 2019; Lamley et al. 2014; Stanek et al. 2016a; Wittmann et al. 2016). Nevertheless, measuring long-range contacts remains challenging due to strong dipolar truncation in broadband recoupling, arising from the dense proton network, and the reduced efficiency of passive mixing schemes at faster spinning rates. To overcome these limitations, pulse sequence development becomes crucial. Tailored pulse sequences can selectively enhance desired correlations while suppressing undesired couplings, thereby complementing the advantages of higher MAS rates. This approach is crucial for making full use of modern MAS probes for biological studies.

The radio frequency-driven dipolar recoupling (RFDR) experiment (Bennett et al. 1992), a mainstay of recoupling at slower MAS rates, has been employed in the detection of long-range ^1^H-^1^H contacts in back-exchanged deuterated proteins (Retel et al. 2017) and fully protonated proteins (Andreas et al. 2016a). However, its efficacy in fully protonated samples is limited by the density of the recoupled proton bath. Various pulse techniques involving selective recoupling schemes and magnetization transfers have been developed to facilitate the detection of long-range contacts in fully protonated samples under very fast MAS frequencies (Nimerovsky et al. 2022; Nimerovsky et al. 2025; Marchand et al. 2022; Duong et al. 2020). Notably, the band-selective spectral spin diffusion spin-lock (BASS-SD) technique, applied at a MAS rate of 111 kHz, enabled the observation of long-range contacts up to 6 Å between protons in the same spectral region in a fully protonated protein (Jain et al. 2017). Recently, symmetry-based recoupling schemes have been exploited in fully protonated samples (Potnuru et al. 2021). In all cases, the challenge for fully protonated samples is achieving broadband recoupling without the abundant proton bath diluting the magnetization to the point where no cross-peaks are observable. Therefore, the development of sensitive pulse sequences in conjunction with fast MAS is a necessary and active field to enhance ^1^H-detected experiments in fully protonated samples.

In a previous study (Friedrich et al. 2020), we introduced two 3D cross polarization (CP) based sequences, (H)NCOH and (H)NCAH (where nuclei in parentheses are used for transfers but not frequency labeled) as a strategy to measure multiple structurally relevant ^1^H–^13^C contacts in a deuterated protein back-exchanged at amide sites. These pulse sequences are built on a train of CP steps, similar to many multidimensional chemical shift assignment experiments (Barbet-Massin et al. 2014; Nieuwkoop et al. 2015; Zhou et al. 2012), with the last polarization transfer step allowed to vary to see weaker long-range couplings. At the moderate MAS rates of 37 and 40 kHz, we successfully observed numerous CO–H^N^ and Cα–H^N^ correlations, revealing structurally significant contacts resulting from hydrogen bonding (Friedrich et al. 2020). In this study, we aimed to determine whether the pulse sequence would yield structurally interesting C-H contacts on a protonated sample at a higher MAS rate. Specifically, we sought to investigate how a dense ^1^H bath of a fully protonated sample impacts the detection of long-range contacts while spinning at 105 kHz. Under these conditions, we successfully observed ^13^C–^1^H contacts from backbone COs and Cαs to multiple H^N^, Hα, and methyl protons to distances up to 6 Å. Since the cross-peaks are observed at the chemical shift of backbone nitrogen and carbons, we were able to establish unambiguous correlations. We see these correlations in both alpha-helix and beta-sheet secondary structures, yielding structurally relevant long-range distance information. Our findings suggest that optimizing the contact times and the radiofrequency (rf) field amplitude of the final C–H CP step using the 1D (HNC)H is essential for obtaining multiple cross-peaks and long-range contacts in the spectral regions of interest.

Unlike the previous study (Friedrich et al. 2020), the observed contacts vary depending on the carbon type, with fewer cross-peaks detected at protonated aliphatic carbon sites relative to naturally unprotonated carbonyl carbons. Thus, this straightforward-to-implement pulse sequence is viable for use with protonated samples with minimal additional optimizations from experiments used for backbone assignments. This is especially helpful for systems where deuteration and back-exchange are impossible, or sensitivity is limited, such as for membrane proteins or samples where the labeled protein is part of a larger complex.

## Materials and methods

### Expression and purification of uniformly ^13^C^15^N-labeled GB1

We freshly transformed GB1 plasmid into Rosetta BL21(DE3)pLysS competent cells. Double colony selection was carried out to select the best-performing colony following the protocol described by Arun and co-workers (Sivashanmugam et al. 2009). A 50 mL 2XYT starter culture was incubated overnight at 37 °C with shaking at 200 rpm and subsequently used to inoculate 100 mL M9-medium prepared following the protocol described by Sharaf and Gronenborn (Sharaf and Gronenborn 2015). The fresh medium was adjusted to a starting optical density of 0.2 and then incubated overnight at 37 °C with shaking at 200 rpm. Cells from this overnight M9 culture were then used to inoculate 1 L M9-medium supplemented with 4 g/L ^13^C-glucose, 1 g/L ^15^NH_4_Cl, 10 mL (10x) ^13^C^15^N BioExpress rich media (Cambridge Isotope Laboratories), 100 mg ampicillin, and other nutrients as specified in the same protocol (Sharaf and Gronenborn 2015). Temperature was maintained at 37 °C, and after two hours of growth, protein expression was induced with 1 mM IPTG. The temperature was reduced to 25 °C for overnight expression.

The cells were harvested at 3900 rpm at 4 °C and resuspended in Phosphate Buffer Saline, PBS (150 mM NaCl and 30 mM KH_2_PO_4_, pH 7.4). Cells were lysed with an LM10 Microfluidizer (Microfluidics) followed by heat treatment at 80 °C to destroy cell matter further. The resulting cloudy solution was spun down at 20,000 rpm at 4 °C for 45 min. The filtered supernatant was loaded onto a pre-equilibrated size exclusion column (ÄKTA pure, Cytiva) with PBS as the sole buffer.

Upon purification, we found that the purified protein solution was contaminated with nucleic acid fragments from UV–visible measurements, which was further confirmed by electrospray ionization mass spectroscopy. Therefore, the solution was further purified by incubating with 5 uL DNA-free benzonase (Sigma Aldrich) and 2 mM MgCl_2_ at 37 °C overnight and then re-purified on the size exclusion column. The isotopic enrichment was confirmed to be 97% using ESI mass spectrometry. Uniformly-labeled GB1 sample was dialyzed thoroughly using a 3.5 kDa MWCO membrane filter against 50 mM NaH_2_PO_4_ buffer (pH 5.5), concentrated to 25 mg/mL, and precipitated in 2:1 volume of 2-methyl-2,4-pentanediol and isopropanol. The microcrystals were incubated in the fridge until use.

The microcrystalline sample was packed into a 0.7 mm rotor with 3D-printed rotor packing tools (Osborn Popp et al. 2023), and all experiments were recorded on an 800 MHz Avance III HD NMR spectrometer. Data processing and peak assignments were done in NMRPipe (Delaglio et al. 1995) and CCPNMR Analysis (Vranken et al. 2005), respectively. Structural representations were prepared using VMD (Humphrey and Schulten 1996). All figures were created in Adobe Illustrator and data processing and analysis was done on NMRbox (Maciejewski et al. 2017).

### CP simulations

The CP transfer efficiency from ^13^C to ^1^H was simulated using the NMR simulation package, Spinach (Hogben et al. 2011) in MATLAB R2024a (MathWorks Inc.) using the experimental MAS NMR conditions. The spin system coordinates were obtained from a protonated version of the GB1 crystal structure (pdb id: 2QMT) and included one structurally relevant ^13^C–^1^H distance in the presence of two shorter range protons. Protons were added to the coordinates deposited in the PDB using the online tool from the Bax group (National Institutes of Health (NIH) 2022. The MAS frequency was fixed at 105 kHz, with the rotor axis oriented at the magic-angle. RF field strengths were 168 kHz on ^1^H and 63 kHz on ^13^C, adjusted to satisfy the Hartman-Hahn match conditions. Contact times were linearly spaced from 0.25 to 12 ms in 24 steps to generate the build-up curves. Powder averaging employed spherical grid with 200 crystalline orientations. All simulations were performed on NMRbox. (Maciejewski et al. 2017)

### NMR measurements

All experiments were recorded on an 800 MHz Avance III HD NMR spectrometer equipped with a 0.7 mm probe with a MAS III spin controller. Experiments were carried out with a VT set point of 283 K at a MAS frequency of 105 kHz (± 5 Hz). In all experiments, 2048 points were acquired in the ^1^H dimension with an acquisition time of 25 ms.

For the (H)NCOH experiment, a contact time of 1.5 ms was applied for the ^1^H–^15^N CP transfer step, optimized to 162 kHz for ^1^H with a rectangular pulse and 67.5 kHz for ^15^N with a tangential ramp of 80–100%. A contact time of 6 ms was used for the N–CO transfer step, optimized to 83 kHz for ^15^N with a rectangular pulse and 22 kHz for ^13^CO with an 80–100% tangential ramp. The contact time for ^13^CO transfer back to ^1^H was optimized for 4 ms, and the applied fields were 167 kHz and 79 kHz for ^1^H and ^13^CO, respectively, with an 80–100% tangential ramp on ^13^CO. 8 scans with an interscan delay of 1.5 s were recorded. The carrier was set to 176.5 ppm and 118 ppm for ^13^CO and ^15^N, respectively. 64 increments were collected in the ^13^CO and the ^15^N dimensions (sweep width 3.5 kHz and 9.1 ms acquisition time). The spectrum was measured in 14 h.

For the (H)NCAH experiment, the carrier was set to 54.7 ppm and 118 ppm for ^13^Cα and ^15^N, respectively. A contact time of 1.5 ms was applied for the ^1^H–^15^N CP transfer step, optimized to 156 kHz for ^1^H with a rectangular pulse and 76 kHz for ^15^N with a tangential ramp of 80–100%. A contact time of 7 ms was used for the ^15^N–^13^Cα transfer step, optimized to 82 kHz for ^15^N with a rectangular pulse and 27 kHz for ^13^Cα with an 80–100% tangential ramp. The contact time for ^13^Cα transfer back to ^1^H was optimized for 4 ms, and the applied field was 160 kHz and 84 kHz for H and Cα, respectively, with an 80–100% tangential ramp on ^13^Cα. 32 scans with an interscan delay of 1.5 s were recorded. 96 increments were collected in the Cα dimension (sweep width 8.75 kHz and 5.5 ms acquisition time). 48 increments were collected in the ^15^N dimension (sweep width 3.5 kHz and 6.9 ms acquisition time). The spectrum was acquired for a total of 62 h. The experimental parameters are summarized in Table S1.

## Results

To investigate whether high MAS rates allow for the detection of long-range contacts in protonated protein samples despite the presence of strong proton-proton homonuclear couplings and short-range proton-carbon heteronuclear couplings, we measured ^13^C–^1^H contacts using two long CP ^1^H detected pulse sequences (Friedrich et al. 2020), (H)NCOH and (H)NCAH, at a MAS rate of 105 kHz. The setup of these experiments is similar to the (H)NCAHA, which can be used for backbone assignment under these conditions (Stanek et al. 2016b), or the suite of (H)CNH assignment experiments, used in both protonated and deuterated samples at high MAS rates (Barbet-Massin et al. 2014; Zhou et al. 2012). Starting with the same CP conditions optimized for the assignment suite, conditions for the long-range (H)NCH experiments can be readily found by increasing the length of the last CP step. The (H)NCOH and (H)NCAH use the same polarization transfer scheme and the pulse sequence for both is given in Fig. S1. Initial polarization transfer is from H^N^ to the directly bonded nitrogen via a short CP period, followed by transfer to the backbone CO in *i-1* position or the Cα of the same residue via specific CP (Baldus et al. 1998). The length of the final polarization transfer step is variable and longer than needed for a one-bond transfer, making it non-specific with transfers to numerous nearby protons from the carbon atom (Friedrich et al. 2020). Therefore, the (H)NCH experiment yields correlations from the backbone Cα or CO to multiple protons in contrast to the (H)CNH experiments, which yield a single peak for each residue.

Cross-peaks were assigned using the chemical shifts from the backbone resonance assignment suites and distances from the crystal structure of the microcrystalline form of GB1 used here (Frericks Schmidt et al. 2007). Additional resonance assignments of sidechain protons in the (H)NCOH spectrum were made by cross-referencing with the complementary (H)NCAH spectrum. The starting carbon was unambiguously determined for most of the residues except a few with degenerate ^15^N and ^13^C chemical shifts. Each plane of the 3D spectra reveals multiple correlations from individual carbon sites to amide and aliphatic protons, with the latter displaying the highest signal intensities.

**Fig. 1:**
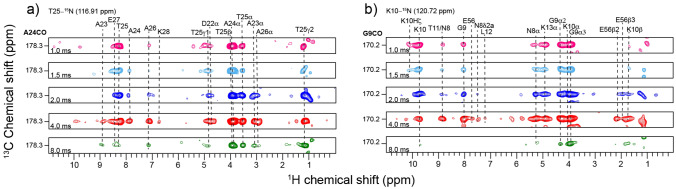
Representative strips of the 3D (H)NCOH spectra of GB1 showing the effect of varying ^13^C–^1^H CP step contact time on cross-peak density. 1 ms, 1.5 ms, 2 ms, 4 ms and 8 ms contact times are depicted in pink, light blue, blue, red and green respectively. (**a**) Shows correlations at the chemical shift of T25N-A24_CO_ to different protons with resonance assignment indicated with dashed lines. Sequential peaks show the highest intensities across all the contact times. At 8 ms, strong correlations are still observed for sidechain protons, but the amide protons show diminished intensities. (**b**) Shows K10N-G9_CO_ correlations to different protons. 4 ms shows the maximum cross-peak density

The rf amplitudes for the spin locks and contact times of the final ^13^C–^1^H CP transfer step are optimized to maximize long-range transfers. Figure 1 compares the peaks observed in the 3D (H)NCOH spectrum as the final contact time was varied from 1 to 8 ms, the value which was optimal in fully deuterated samples. Figure 1a shows correlations from a representative residue, A24_CO_, to amide and sidechain protons of residues across different contact times in the (H)NCOH experiment. The sequential peaks corresponding to T25-H^N^ and A24-Hα are consistently observed with similar intensity at all contact times. As the contact time increases to 4 ms, additional cross-peaks can be assigned to both sequential residues as well as structurally defining contact to K28. However, at 8 ms, fewer cross-peaks are observed, especially in the amide spectral region. This observation contrasts with similar experiment conducted on deuterated samples at 37 kHz, where mixing times up to 8 ms continued to show additional cross-peaks. This drop in cross-peak intensity at longer mixing times is likely due to ^1^H–^1^H spin diffusion during these longer spin locks (Kolodziejski and Klinowski 2002). We see similar behavior for a beta-sheet residue G9_CO_ in Fig. 1b, with additional proton contacts as contact time increases up to 4 ms, and a sharp decrease, especially in the amide region at a contact time of 8 ms. Based on these results, the contact time for the final CP step was set to 4 ms for all subsequent acquisitions of both (H)NCOH and (H)NCAH spectra.

**Fig. 2:**
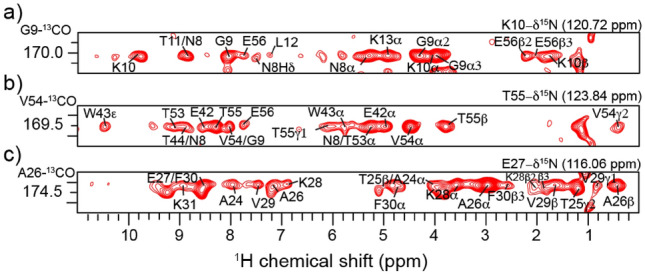
^13^CO–^1^H 2D planes of the 3D (H)NCOH spectrum. Each strip contains assignments of the observed ^13^CO–^1^H correlations, including amide and aliphatic correlations. (**a**) shows G9_CO_ correlations to protons of nearby residues, T11, N8, L12, K13, and also the long-range, E56. (**b**) V54_CO_ also shows multiple proton cross-peaks correlations to neighboring residues and to E42 and N8. (**c**) A26_CO_ shows correlations to A20, A24, T25, A26, E27, K28, V29, F30 and K31

**Fig. 3:**
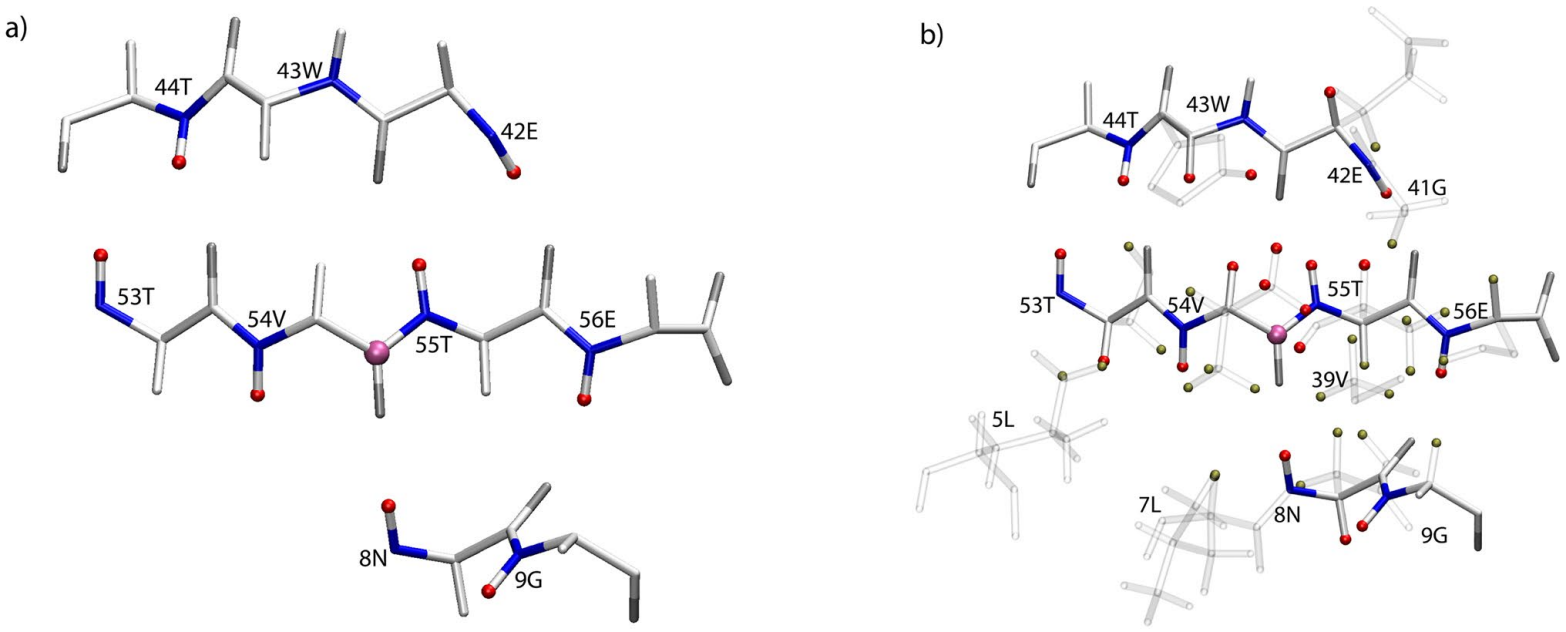
Structural representation of V54_CO_ contacts. Oxygen, Nitrogen and carbon atoms are represented in gray, blue and white respectively. V54_CO_ atom is depicted with a mauve sphere. (**a**) Observed amide protons are depicted with red spheres. (**b**) Additional aliphatic protons are depicted with red spheres. Tan spheres depict protons within 6 Å not observed

The (H)NCOH spectrum with a contact time of 4 ms revealed a range of H^N^ and aliphatic correlations that provide valuable insights into residue-specific interactions and structural connectivity (Fig. 2). For example, for 9G_CO_ (Fig. 2a), H^N^ correlations are observed to E56, L12, T11, G9, and the sequential K10. Moreover, there is a correlation to the sidechain N8-Hδ. Similarly, at the V54_CO_ frequency (Fig. 2b), there are H^N^ correlations to E42, G9, T44, T53, T54, E56, T55, and its hydrogen bonding partner, N8. Resolved aliphatic correlations, mostly Hα, Hβ and Hγ, were made across the spectrum, especially to the same residues observed in the H^N^ region. For example, V54_CO_ (Fig. 2b) shows Hα and Hγ correlations to T55, V54, T53, N8, E42, W43. V54_CO_ also shows correlations to W43-Hε (Fig. 2b); this side chain nitrogen was extremely important for defining the core of GB1 in the first proton-detected structure of GB1^1^. Figure 3 displays the protons within 6 Å of V54_CO_ to better visualize the correlations. We can see that the density of side chain protons is more important than the existence of hydrogen bonding in explaining the appearance of cross peaks.

**Fig. 4:**
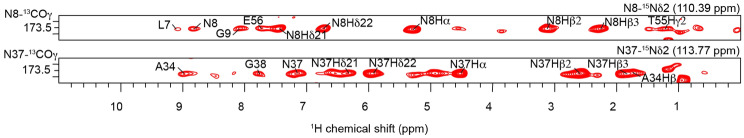
^15^Nδ–^13^Cγ 2D planes illustrating the observed ^13^CO–^1^H correlations from asparagine sidechain. The N37_Cγ_ exhibits correlations exclusively to protons of residues in close proximity, while the N8_COγ_ shows correlations to protons of nearby residues and more distant residue, E56

To understand the completeness of CO–H^N^ correlations observed in the 3D (H)NCOH spectrum, the protonated GB1 crystal structure (Institute and of Health (NIH) 2022; Frericks Schmidt et al. 2007), was analyzed to identify atoms within defined distances. For distances ≤ 3 Å, we observed 97% of expected correlations which were almost exclusively intraresidue (*i* = *j*) or sequential *(|i − j|*= *1*) residues. Of the amide correlations expected between 3–4 Å, 81% were observed, and 43% of these are medium (1 <|*i − j*| ≤ 5) to long-range peaks (|*i − j*| ≥ 5), with the rest again sequential. Most of these long-range correlations are the result of the hydrogen bonding network fundamental to the formation of protein secondary structure, namely across adjacent beta strands and to the H_*i*+*4*_ position in the alpha-helix. Also, between 4–6 Å, ~ 39% of observed peaks are medium to long-range correlations, while 61% are sequential. The summary statistics of observed peaks is shown in Table S2. In addition, two sidechain carbonyl carbons, 8N_Cγ_ and 37N_Cγ_, show multiple proton cross-peaks (Fig. 4). 8N_Cγ_ shows correlations to L7, G9, E56 and N8, while 37N_Cγ_ shows correlations to A34, G38 and N37. About 203 H^N^ cross-peaks were unambiguously assigned in the (H)NCOH spectrum. A full list of manually assigned correlations in the (H)NCOH spectrum is listed Table S3. Additional strips from the spectrum and sidechain correlations in glutamine sidechain CO are shown in Fig. S3 and S4 respectively.

**Fig. 5:**
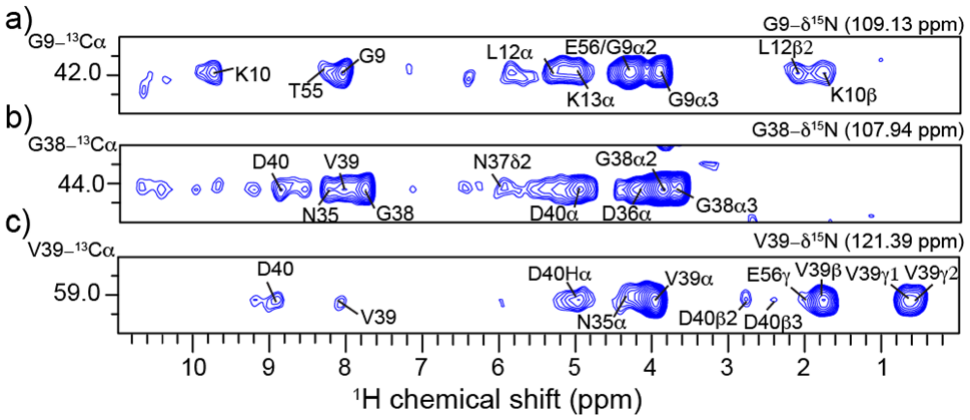
^15^N–^13^Cα 2D planes of the 3D (H)NCAH spectrum. Each strip displays the assigned ^13^Cα–^1^H correlations, including amide and aliphatic correlations. Residue G38 shows more cross-peaks in the amide region compared to G9 and V39. Each plane contains cross-peaks for its own protons

**Fig. 6:**
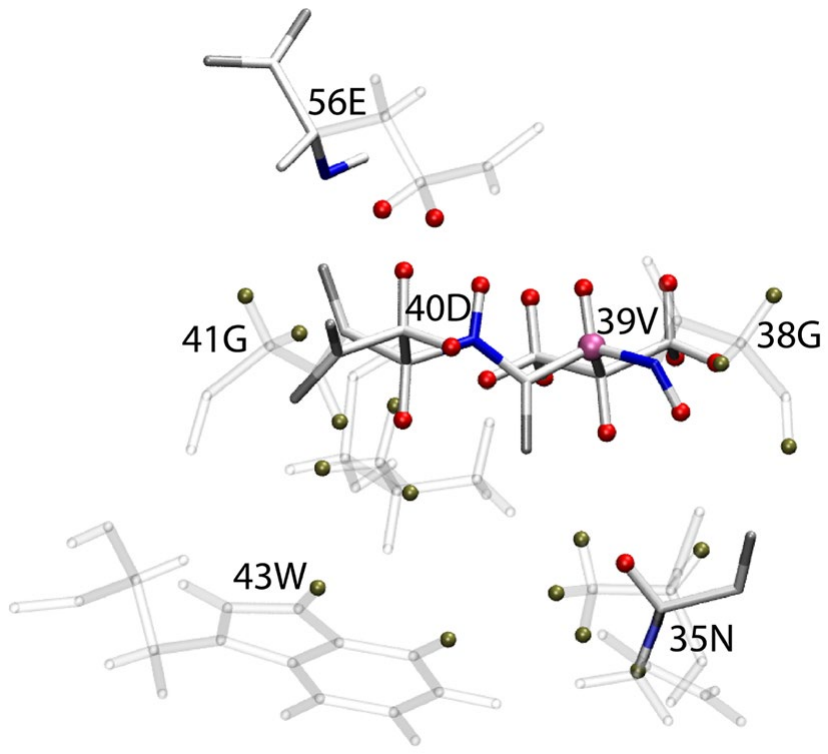
Structural representation of V39_Cα_ contacts. Oxygen, Nitrogen and carbon atoms are represented in gray, blue and white respectively. V39_Cα_ atom is depicted with a mauve sphere. Observed hydrogen atoms are depicted with red spheres. Tan spheres depict other neighboring protons not observed at 6 Å

The primary distinction between the observed correlations in the (H)NCOH and (H)NCAH spectra lies in the distribution of intraresidue, sequential, and long-range peaks. The (H)NCOH spectrum gives a range of medium and long-range cross-peaks, reflecting its capabilities of capturing interactions beyond direct neighbors. In contrast, the (H)NCAH spectrum predominantly displays sequential cross-peaks within a single residue or immediate neighbors. Most of the cross-peaks observed in the (H)NCAH spectrum correspond to aliphatic protons, with comparatively fewer correlations to H^N^ protons. Typically, only the H^N^ correlated with the Cα of a given residue is detected. However, the weaker cross-peaks in the amide region were observed with extended signal averaging (62 h experiment time, ~ 3.5 × longer than the CO version). For instance, at the frequency corresponding to the G9_Cα_ chemical shift (Fig. 5a), there is an H^N^ correlation to T55 in addition to the sequential residue K10. Also, at G38_Cα_ (Fig. 5b), H^N^ correlations are observed with the neighboring residues N37, V39, D40, and N35. Additionally, aliphatic correlations were detected for N37 and D40 alongside the expected correlations to G38. Finally, for V39_Cα_, the H^N^ correlation is only to D40 and V39 (Fig. 5c, 6). A full list of manually assigned observed correlations is listed in Table S4. Notably, some of these correlations correspond to a distance greater than 6 Å, such as the G9_Cα_–T55H^N^ and G38_Cα_–N35H^N^ correlations, indicative of spin diffusion which will be discussed more later.

The (H)NCAH experiment has only ~ 67% of the sensitivity of the (H)NCOH experiment (Fig. 6), a difference mainly due to lower N-CA CP efficiencies which we also observe in our assignment spectra at 105 kHz MAS. Both experiments yielded C-H correlations, with the (H)NCOH experiment producing CO–H correlations in both the amide and sidechain regions. In contrast, fewer Cα–H^N^ contacts were observed in the (H)NCAH experiment, with most contacts localized in the intraresidue. Since this was a protonated sample, stronger correlations were observed across both experiments for the aliphatic spectral region. The observed correlations to Hα and methyl protons were sufficient to allow for the assignment of sidechain protons in GB1. Although sidechain proton assignments were established in a study by Stanek and co-workers (Stanek et al. 2016b), the value of this experiment lies in the additional correlations observed with other sidechain protons, providing structurally relevant contacts. This experiment could be used in conjunction with (H)CCH-type experiments, which provide comprehensive sidechain proton information (Stanek et al. 2016b).

As shown in Fig. 6, the 1D (HNC)H version of the experiment (the first row of the 3D) gives valuable information about the eventual content of the 3D experiments. As such, it was used extensively to optimize the final CP step. While, in general, spin lock power levels based on the optimal values from (H)CNH-type experiments were close to the final conditions used for the (H)NCH experiments, we would like to note an intriguing dependence on the position of the tangent ramp of the final CP. As the (H)NCAH experiment did not initially yield large numbers of long-range contacts, we systematically sampled a wide array of potential Cα-H cross polarization conditions. We have previously noted that for 2D (H)NH and (H)CH-type experiments, having a variable ramp on the ^1^H channel (i.e., the destination nucleus) during the return ^15^N–^1^H or ^13^C–^1^H CP step results in maximal signal for short, one-bond CP transfers (data not shown). This effect is small, often around 10%, but notable enough that we include this optimization beyond the symmetrical condition where the initial and return CP steps (H–N and N–H) are identical (Matsunaga et al. 2021). For the longer-range CP used in this study, we found that the position of the ramp in the final CP step affected which types of cross-peaks were observed in the experiment. Fig. S2 shows the results of optimizing the total signal intensity of the (HNCO)H 1D with the ramp on the ^1^H (green) and ^13^C (red). Here, we see an almost complete lack of ^13^CO–^1^Hα transfer with the ramp on the ^1^H. Larger cross-peak densities were observed when the ramp is on ^13^C compared to when placed on ^1^H. Given our desire to detect structurally relevant CO–H^N^ correlations, we were hopeful that this “no Hα” condition might yield additional useful cross peaks in the H^N^ region. However, as Fig. S2b shows, if anything, there were fewer long-range correlations in the ^1^H ramped spectrum. Given that our ^1^H carrier was on the water signal at 4.6 ppm, we imagine there could be some on resonance band selective ^1^H–^1^H mixing occurring with the ^1^H ramp (à la BASS-SD). However, given the high powers (160 kHz), a quick calculation confirms that the relative power for the CP match conditions should not be notably different due to the few kHz offset. In fact, arrays of the ^13^C power level without ramps (rectangle to rectangle) confirm the C–H selectivity is a ^13^C driven phenomenon, with the C–H^N^ and C–Hα transfers happening at slightly different carbon spin lock fields.

**Fig. 7:**
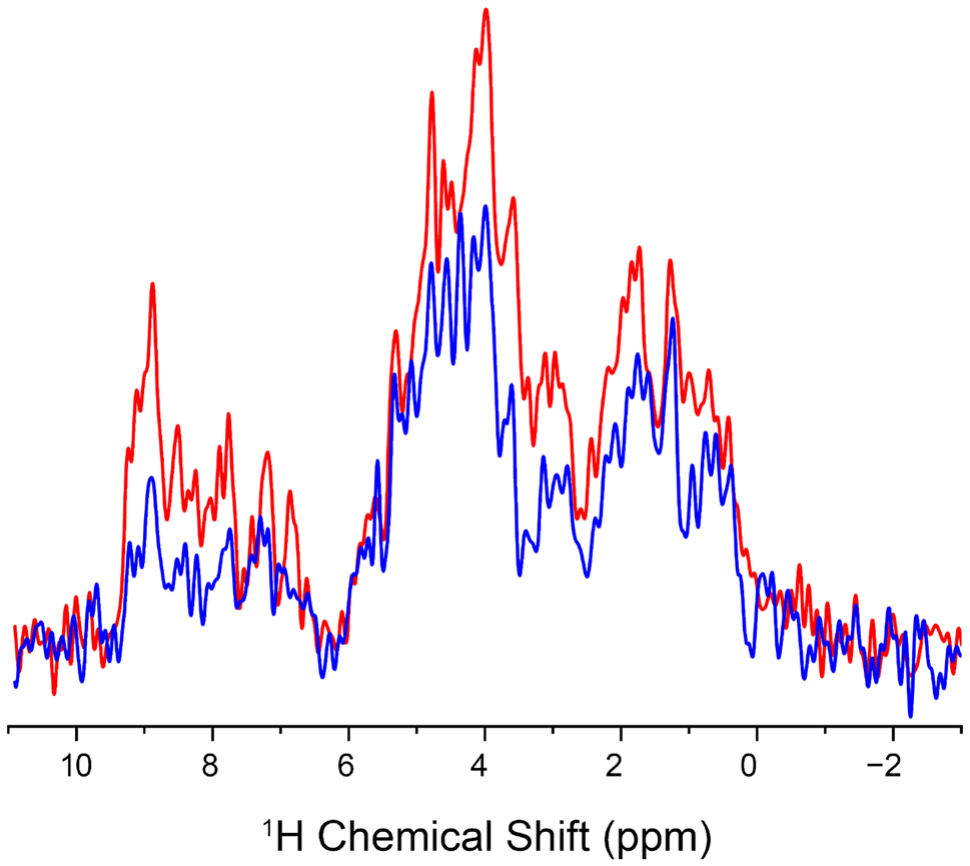
^1^H 1D overlay of (H)NCOH (*red*) and (H)NCAH (*blue*) spectra highlighting differences in sensitivity

Having thoroughly explored the parameter space for the long-range ^13^C–^1^H CP, we concluded that additional proton on the Cα was preventing longer range peaks from being observed. To support this hypothesis of ‘dipolar truncation’ we turned to spin physics simulations using the program Spinach (Hogben et al. 2011). We chose the V54-N8 hydrogen bond as observed in Fig. 2 and 3 as our test case. Fig. S5 shows the CP build ups for V54CO and T55Cα to N8 H^N^ in the presence of T55Hα and H^N^. The build-up for the CO–H^N^ peak is 10 × stronger than the Cα–H^N^ cross peak, despite being less than 1 Å closer. The rapid transfer of magnetization from the Cα to the attached Hα (100 × stronger than the long-range H^N^ at 1 ms) hinders the transfer of magnetization to the more interesting long-range peak, N8. While the long-range hydrogen bonding interaction simulated here had reasonable build up rates (Fig. S5a), it is also clear that the buildup is much faster for the nearby T55Hα and T55H^N^ protons (Fig. S5b and c). This leads us to the second mechanism we propose for the appearance of long-distance cross peaks: spin diffusion. We are using the generic term spin diffusion to describe what might be an active (HORROR style) ^1^H–^1^H recoupling during the final CP step, or a passive ^1^H–^1^H mediated mixing during the time spent on the Z-axis during the MISSISSIPPI solvent suppression. Thus, for spin systems like V39 (Fig. 5c, 7) with strongly coupled proton networks, we observe the long-distance V39Cα-Hγ1/2 cross peaks as the strongest in the spectrum as well as V39Cα-D40Hβ2/3 and even V39Cα-E56Hγ, a distance of 8 Å, while no corresponding peaks to the neighboring G38, which lacks a large protonated sidechain, are seen. Others have shown that these tight aliphatic-aliphatic contacts could be useful in the context of protein structure determination, especially with the use of a 4th dimension to help resolve ambiguity in the second proton dimension (Huber et al. 2011).

## Discussion and conclusions

We have demonstrated that long-range ^1^H–^13^C cross polarization can be used to detect structurally relevant contacts in fully protonated proteins at 100 + kHz MAS. Contacts between carbons and nearby protons were observed using the (H)NCOH and (H)NCAH 3D experiments. In contrast to what was observed in deuterated samples at slower spinning, the distribution of the observed correlations differed for the CO and Cα-based experiments, with the protonated Cα causing more sequential transfers to the detriment of longer-range H^N^ contacts. While spin diffusion in the sidechains allowed for some longer-range correlations (similar to those observed in band selective mixing), this transfer pathway decreased CO–H^N^ correlations at longer CP times. Protonated side chains do yield some structurally relevant side chain-CO peaks, which would help define side-chain orientations and help with the problem of very long H^N^–H^N^ distances in helical bundles.

In practical terms, the heteronuclear nature of the mixing in this pulse sequence means that the (H)NCH 1D experiments are an effective proxy for the appearance of long-range peaks. This was most notable in the (H)NCAH, where careful optimization of the Cα–H condition can give a larger proportion of amide peaks in the 1D, which corresponds to more long-range peaks in the 3D, likely due to avoiding conditions with too much ^1^H–^1^H spin diffusion. Overall, this experiment is easy to set up using conditions from existing (H)CNH-type assignment spectra, making it a powerful tool for gaining information on sidechains and some structural restraints on protein samples with lower sensitivity, for which many optimizations are not practical. As many of the most biologically interesting samples exist at the edge of the practical sensitivity limit, including samples where the protein of interest is a minor component, such as membrane-bound proteins or in-cell NMR, a straightforward experiment for measuring distances without extensive optimization could prove valuable.

## Supplementary Information

Below is the link to the electronic supplementary material.

Supplementary file1 (DOCX 1431 KB)

## Data Availability

No datasets were generated or analysed during the current study.
