# Correction: Measuring long-range contacts in a fully protonated protein at 105 kHz magic angle spinning

**DOI:** 10.1007/s10858-025-00478-7

**Published:** 2025-11-21

**Authors:** Zainab O. Mustapha, Eren H. Ozturk, Benjamin E. Lefkin, Diana Grajeda, Andrew J. Nieuwkoop

**Affiliations:** Department of Chemistry and Chemical Biology, Rutgers, The State University of New Jersey, Piscataway, NJ 08854 USA

**Correction to: Journal of Biomolecular NMR** 10.1007/s10858-025-00477-8

In this article the caption to Fig. 6 was inadvertently appeared as “Structural representation of V39Cα contacts. Oxygen, Nitrogen and carbon atoms are represented in gray, blue and white respectively. V39Cα atom is depicted with a mauve sphere. Observed hydrogen atoms are depicted with red spheres. Tan spheres depict other neighboring protons not observed at 6 Å 1H 1D overlay of (H)NCOH (red) and (H)NCAH (blue) spectra highlighting differences in sensitivity”, but should have appeared as “Structural representation of V39Cα contacts. Oxygen, Nitrogen and carbon atoms are represented in gray, blue and white respectively. V39Cα atom is depicted with a mauve sphere. Observed hydrogen atoms are depicted with red spheres. Tan spheres depict other neighboring protons not observed at 6 Å”.

In this article the caption to Fig. 7 was inadvertently appeared as “Structural representation of V39Cα contacts. Oxygen, Nitrogen and carbon atoms are represented in gray, blue and white respectively. V39Cα atom is depicted with a mauve sphere. Observed hydrogen atoms are depicted with red spheres. Tan spheres depict other neighboring protons not observed at 6 Å”, but should have appeared as “^1^H 1D overlay of (H)NCOH (red) and (H)NCAH (blue) spectra highlighting differences in sensitivity”.

The original article has been corrected.

