# Supplementary material for: Measuring long-range contacts in a fully protonated protein at 105 kHz magic angle spinning
Source: J Biomol NMR. 2025 Sep 26;79(4):331–9. doi: 10.1007/s10858-025-00477-8 (PMC12664857; doi:10.1007/s10858-025-00477-8)
Supplement: Supplementary file 1 — Supplementary file1 (DOCX 1431 KB) [file 10858_2025_477_MOESM1_ESM.docx]

**Supplementary Information**

Measuring Long-Range Contacts in a Fully Protonated Protein at 105 kHz Magic

Angle Spinning

Zainab O. Mustapha, Eren H. Ozturk, Benjamin E. Lefkin, Diana Grajeda, Andrew J. Nieuwkoop*

Department of Chemistry and Chemical Biology, Rutgers, The State University of New Jersey, Piscataway, New Jersey 08854, United States

*Correspondence to: an567@rutgers.edu


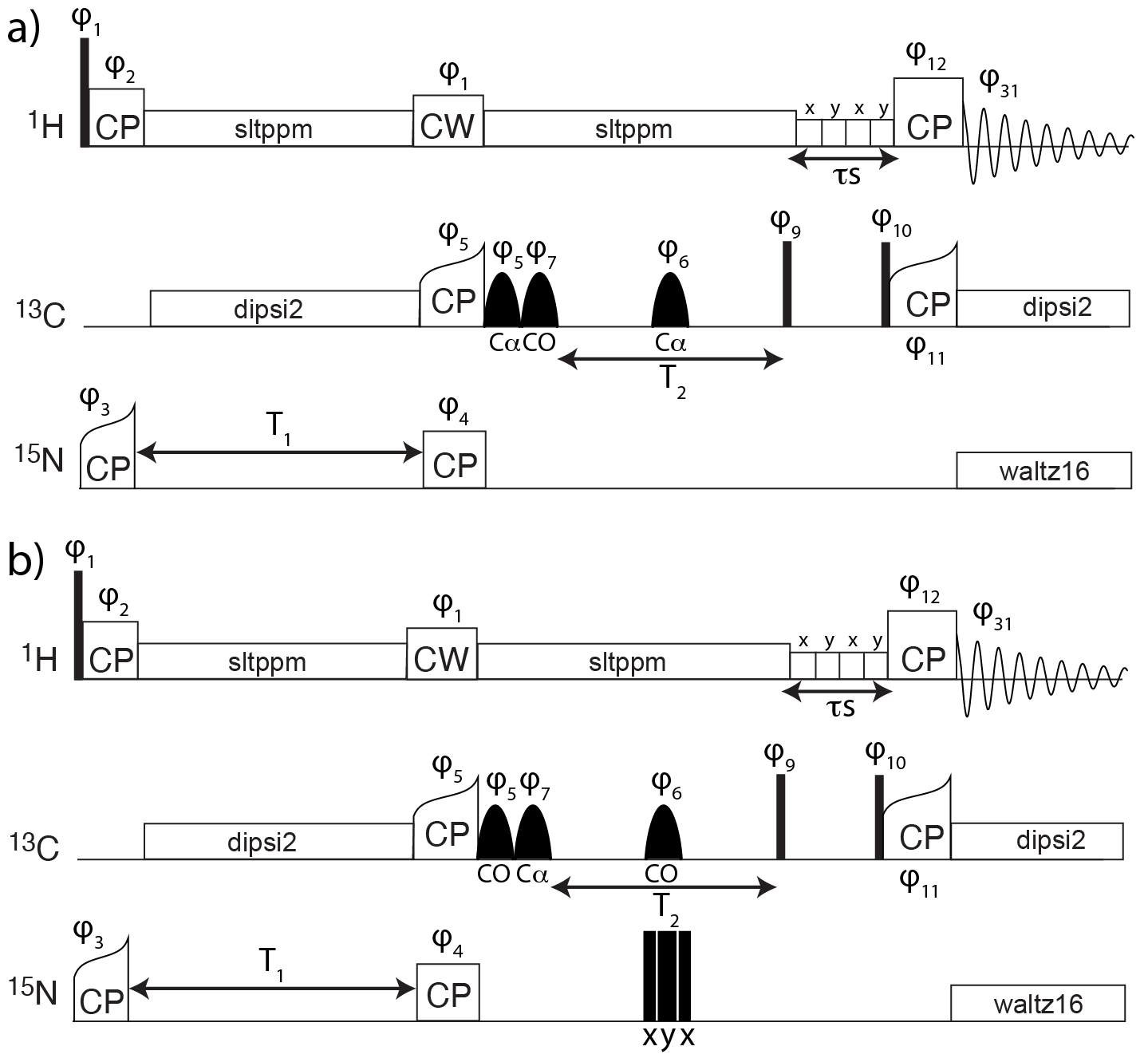


**Fig. S1**: Pulse sequence for detecting ^13^C-^1^H contacts. The ^13^C carrier frequency is set to allow the acquisition of (a) (H)NCOH and (b) (H)NCAH. Constant decoupling duty cycles were used during the t_1_ and t_2_ periods. Wide and narrow black rectangle indicate π and π/2 pulses respectively. Filled bell shapes depicts selective inversion pulses. CP cross polarization, CW, continuous wave decoupling, τ_S_, MISSISSIPPI solvent suppression. The phase cycle is as follows: ϕ_1_ =1133, ϕ_2_ =0, ϕ_3_ =1, ϕ_4_ =1, ϕ_5_ =0, ϕ_6_ =0, ϕ_7_ =01, ϕ_9_ =1, ϕ_10_ =0, ϕ_11_ =1, ϕ_12_ =0, ϕ_rec_ = 1331

Table S1: Acquisition parameters for 3D (H)NCOH & (H)NCAH spectra acquired on microcrystalline GB1 on 18.8 T magnetic field (^1^H frequency = 800 MHz), VT set point: 283 K. The reported field strength for ramped pulses corresponds to the maximum value at the top of the ramp.

| Experiment | | |
| --- | --- | --- |
|  | (H)NCOH | (H)NCAH |
| ^1^H offset | 4.6 ppm | 4.6 ppm |
| ^13^C offset | 176.5 ppm | 54.7 ppm |
| ^15^N offset | 118 ppm | 118 ppm |
| 1^st^ CP (HN) | | |
| ^1^H field | 162 kHz | 155 kHz |
| ^15^N field | 76 kHz | 75 kHz |
| ^15^N shape | TAN_20D_4B.wave | TAN_20D_4B.wave |
| Contact time | 1.5 ms | 1.5 ms |
| 2^nd^ CP (NC) | | |
| ^15^N field | 83 kHz | 81 kHz |
| ^13^C field | 23 kHz | 27 kHz |
| ^15^N shape | RECT.250 | RECT.250 |
| ^13^C shape | TAN_15D_4B.wave | TAN_15D_4B.wave |
|  |  |  |
| Contact time | 6.0 ms | 7.0 ms |
| 3^rd^ CP (CH) | | |
| ^13^C field | 79 kHz | 86 kHz |
| ^1^H field | 168 kHz | 158 kHz |
| ^13^C shape | TAN_20D_4B.wave, No H ramp | TAN_20D_4B.wave, No H ramp |
| Contact time | 4.0 ms | 4.0 ms |
| Decoupling | | |
| waltz16 (^15^N Field) | 5 kHz | 5 kHz |
| dipsi2 (^13^C Field) | 10 kHz | 10 kHz |
| slTPPM | 26 kHz | 25 kHz |


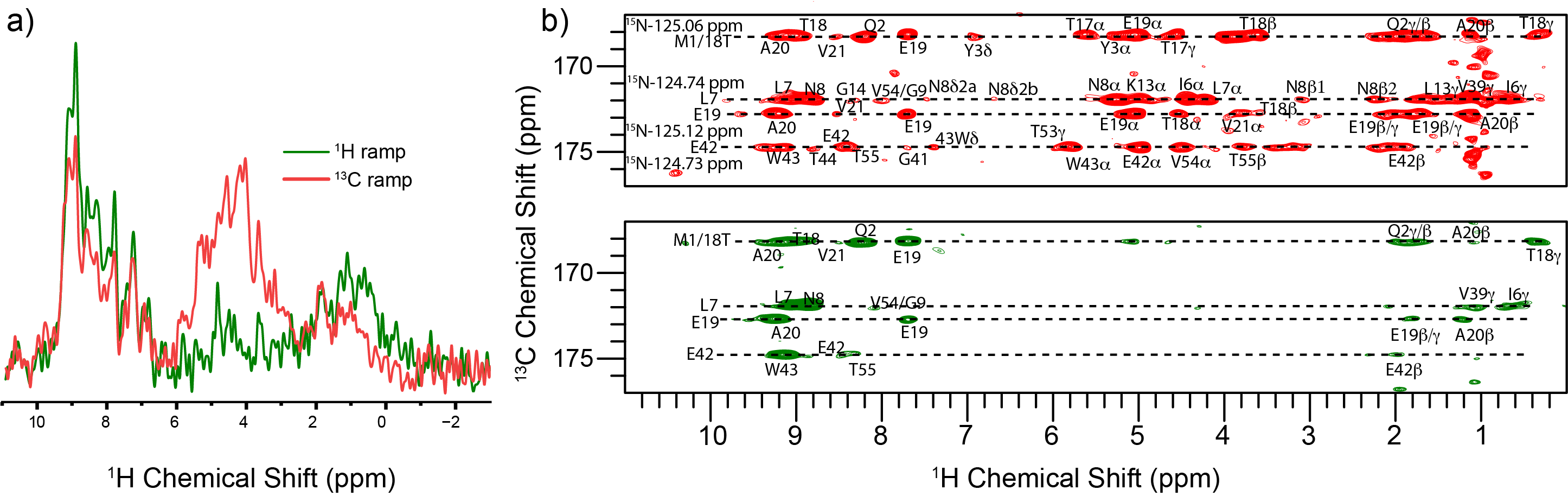


**Fig. S2** Representation of (H)NCOH at different CP ramp conditions. (a)1D ^1^H spectra of (H)NCOH with ramp on ^1^H (green) and ramp on ^13^C (red) (b) 3D planes of (H)NCOH showing effects of the different ramp conditions. Green spectrum has diminished cross-peak density


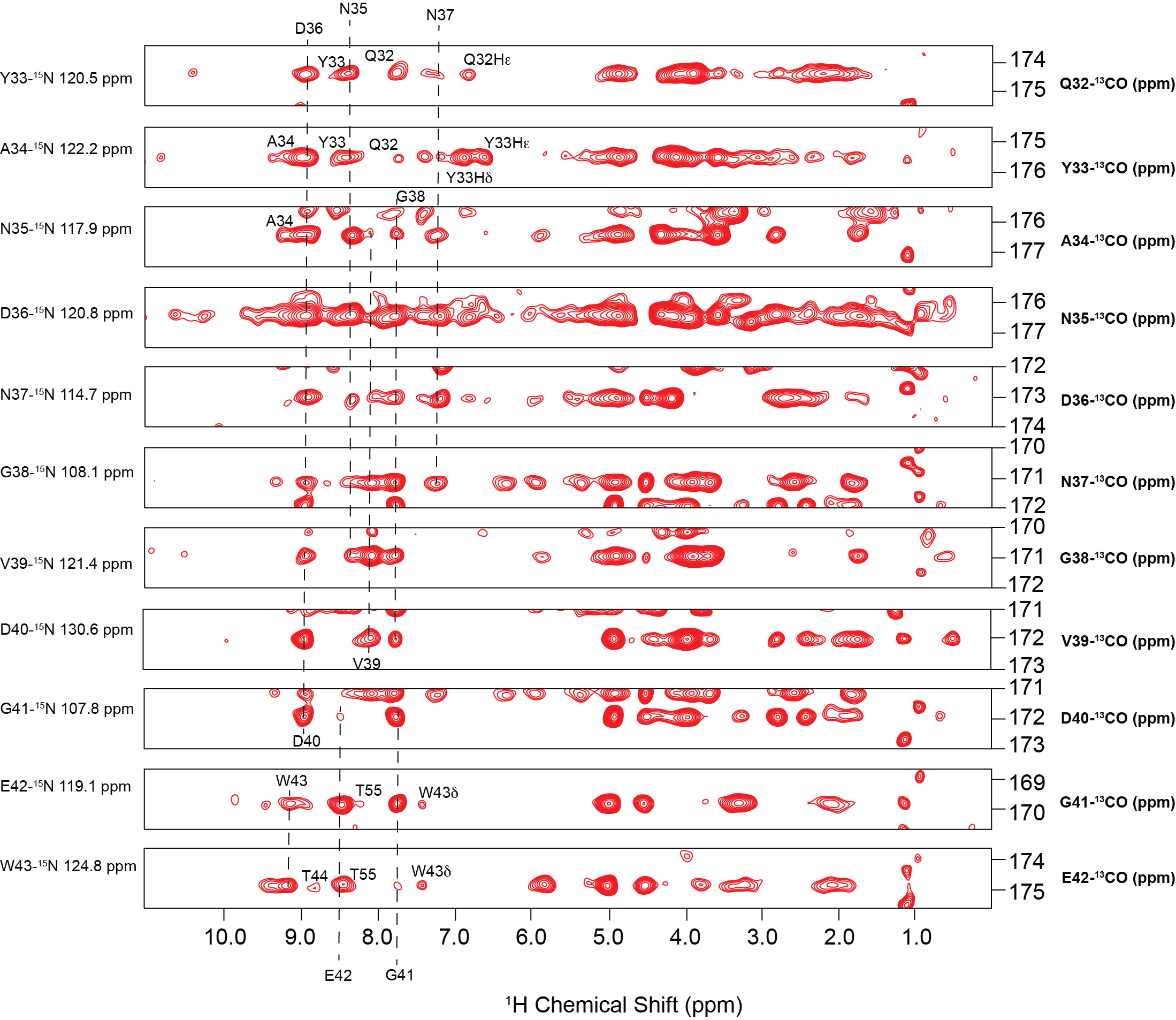


Fig. S3: Representative strips from (H)NCOH experiment showing observed correlations with minimal annotations.


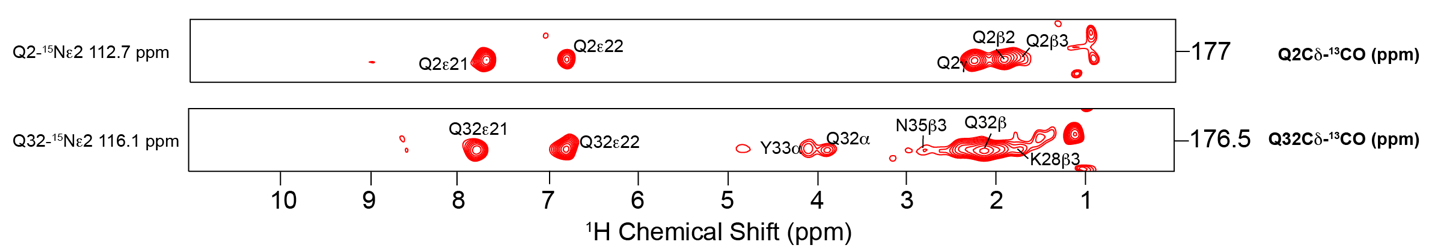


Fig. S4: Observed correlations from Glutamine NCO sidechain.


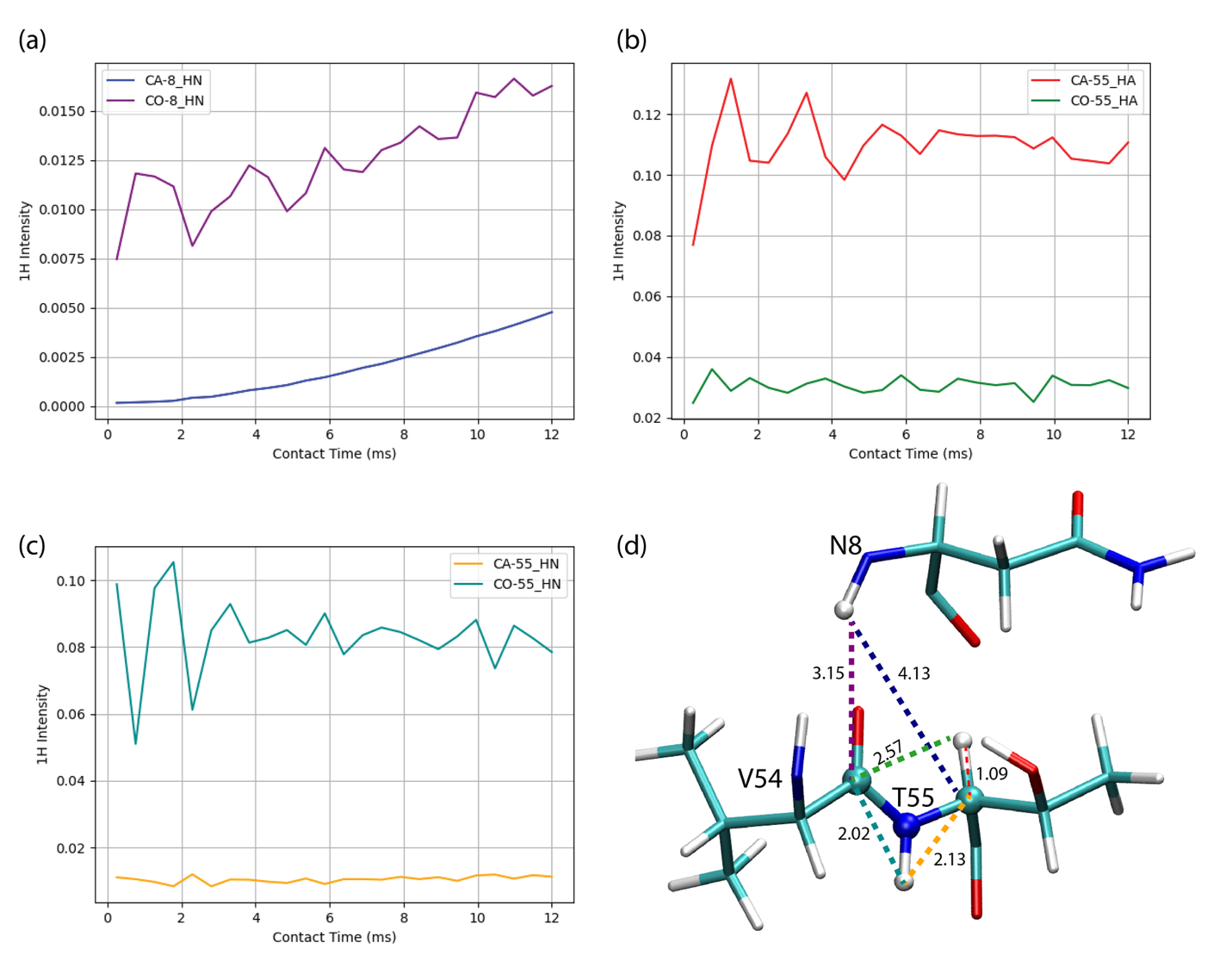


Fig. S5: Simulations of V54CO and T55Cα to 8 H^N^, 55Hα and 55H^N^. (a) build-up curves for both V54CO and T55Cα to 8H^N^ in the presence of the two other protons. (b) build-up curves for both V54CO and T55Cα to T55Hα in the presence of the two other protons. (c) build-up curves for both V54CO and T55Cα to T55H^N^ in the presence of the two other protons. (d) representation of simulated spin systems. Mauve, blue, red and white sticks represents carbon, nitrogen, oxygen and hydrogen atoms. The atoms directly involved are represented as spheres. Contacts are colored in accordance with the curves they represent and the respective distances is indicated. All distances are in Angstrom.

Table S2: Summary of observed cross-peaks in the (H)NCOH and (H)NCAH experiments (amide and aliphatic contacts) without considering any overlapping peaks

|  | (H)NCOH | (H)NCAH |
| --- | --- | --- |
| Intraresidue (*i=j*) | 172 | 226 |
| Sequential (\|*i-j*\| =1) | 196 | 65 |
| Medium range (1< \|*i-j*\| ≤ 5) | 138 | 20 |
| Long range (\|*i-j*\| ≥ 5) | 57 | 13 |
| Hydrogen bonds* | 20 | - |
| Total (*excluded) | 563 | 324 |

Table S3: Full-list of observed cross-peaks in the (H)NCOH spectrum

| Residues | Observed Correlations | | | | | | | |
| --- | --- | --- | --- | --- | --- | --- | --- | --- |
| Q2M1 | G2-HN | V21-HN | Y3Hε | V21-H𝛼 | Q2-Hβ2 | G2-HN | E19-Hβ2 |  |
| K4Y3 | K4/L5-HN | F30-Hz | T17-H𝛼 | K4-H𝛼 | Y3-Hβ3 |  |  |  |
| L5K4 | K4/L5-HN | E15-H𝛼 | K4-H𝛼 | T18-Hβ | K4-H𝛾 | L5-H𝛿 |  |  |
| L7I6 | N8/I6-HN | L7/L5-HN | V54-HN | E15-H𝛼 | N8-H𝛼 | I6-H𝛼 | L7-H𝛼 | I6-Hβ |
|  | V54-H𝛾2 | L7-Hβ3 |  |  |  |  |  |  |
| N8L7 | L7-HN | N8-HN | G14-HN | V54/G9-HN | N8-H𝛿21 | N8-Hβ3 | N8-H𝛼 | L7-H𝛼 |
|  | N8-H𝛿22 | V39-H𝛾2 | L5-H𝛿 | N8-Hβ2 |  |  |  |  |
| G9N8 | K10-HN | L7-HN | T11-HN | N8-HN | G9-HN | T55-HN | N8-H𝛿21 | N8-H𝛿22 |
|  | N8-Hβ3/E56-Hβ2 | N8-Hβ2 | L7-H𝛼/G9-H𝛼2 | G9-H𝛼3/K10-H𝛼 | T55-H𝛾2 | E56-Hβ3 |  |  |
|  | N8-H𝛼 | K13-H𝛿 | L7-Hβ2 |  |  |  |  |  |
| K10G9 | K10-HN | T11/N8-HN | G9-HN | E56-HN | L12-HN | K10-H𝛼 | N8-H𝛼 | K13-H𝛼 |
|  | E56-Hβ2 | N8-H𝛿2a | K10-Hβ | T55-H𝛾2 | E56-Hβ3 | G9-H𝛼3 | G9-H𝛼2 |  |
| T11K10 | K10-HN | T11/N8-HN | G9-HN | L12-HN | K10-H𝛼 | G9-H𝛼2 | K10-Hβ |  |
| L12T11 | L12-HN | K10-HN | T11-HN | K13-HN | L12-H𝛼 | T11-H𝛼 | T11-Hβ | K10-Hβ |
|  | T11-H𝛾2 | G9-H𝛼2 |  |  |  |  |  |  |
| K13L12 | K10-HN | T11-HN | L12-HN | K13-HN | L12-H𝛼 | K13-H𝛼 | G9-H𝛼3 | K13-Hβ2 |
|  | K13-H𝛾2 | K13-H𝛿 | T11-H𝛾2 | L7-H𝛿 | K13-Hβ3 |  |  |  |
| G14K13 | L7-HN | K13-HN | G14-HN | L12-HN | E15-H𝛼 | L12-H𝛼 | K13-H𝛼 | I6-H𝛼 |
|  | L7-H𝛼 | L7-H𝛿 | K13-H𝛿2 | K13-Hβ2 | G14-H𝛼2 |  |  |  |
| E15G14 | L7-HN | E15-HN | T16-HN | E15-H𝛼 | G14-H𝛼3 | L7-H𝛼 | I6-H𝛼 | G14-H𝛼2 |
|  | E15-Hβ2 |  |  |  |  |  |  |  |
| T16E15 | T16-HN | T17-HN | L5/L7-HN | T16-Hβ | I6-H𝛼 | L7-H𝛿 | G14-H𝛼3 |  |
|  | E15-H𝛼/T16-H𝛾1 |  |  |  |  |  |  |  |
| T17T16 | T16-HN | T17-HN | L7-H𝛿 | T16-H𝛼 | T18-Hβ | T17-H𝛼 |  |  |
|  | T18/L5-HN | T16-H𝛾1 |  |  |  |  |  |  |
| T18T17 | T17-HN | T18/L5-HN | E19-HN | E19-H𝛼 | T16-H𝛼 | T17-Hβ | T18-Hβ | T17-H𝛼/T18-H𝛾1 |
|  | K4-H𝛼 | T18-H𝛾2 |  |  |  |  |  |  |
| E19T18 | T18-HN | E19-HN | A20-HN | T18-H𝛾1 | T18-H𝛼 | E19-H𝛼 | T18-H𝛾2 | T18-Hβ |
| A20E19 | A20-HN | V21-HN | E19-HN | E19-H𝛼 | T18-H𝛼 | V21-H𝛼 | E19-Hβ2 | E19-Hβ3 |
|  | V21-H𝛾1 |  |  |  |  |  |  |  |
| V21A20 | E19-HN | A20-HN | V21-HN | D22-HN | E19-H𝛼 | V21-H𝛼 | V21-H𝛾1 | E19-Hβ2 |
| D22V21 | A20-HN | V21-HN | D22-HN | V21-H𝛼 | A20-Hβ | D22-Hβ2 | V21-H𝛼 |  |
|  | V21-H𝛾1 | D22-Hβ3 | T25-H𝛾1 |  |  |  |  |  |
| A23D22 | D22/A26-HN | A24-HN | T25-HN | A23-HN | T25-H𝛾2 | V21-H𝛼 | A23-H𝛼 |  |
|  | T25-H𝛾2 | A26-H𝛼 |  |  |  |  |  |  |
| A24A23 | A24-HN | D22/A26-HN | K28-HN/Y3H𝛿 | T25-HN | A23-HN | E27-HN | T25-H𝛾2 | A23-H𝛼 |
|  | A24-H𝛼 | T25-H𝛼 | T25-H𝛾1 |  |  |  |  |  |
| T25A24 | A23-HN | E27-HN | T25-HN | A24-HN | A26-HN | K28-HN | T25-H𝛾1 | T25-H𝛼/K28-H𝛼 |
|  | A23-H𝛼 | A26-H𝛼 | T25-H𝛾2 | A24-H𝛼/T25-Hβ | D22-H𝛼 |  |  |  |
| A26T25 | T25-HN | A24-H𝛼/T25-Hβ | K28-HN | A24-HN | T25-H𝛾1 | A26-HN | T25-H𝛼 | A26-H𝛼 |
|  | A24-Hβ | E27-H𝛼 | A23-Hβ | A26-HN |  |  |  |  |
| E27A26 | K31-HN | E27/F30-HN | A24-HN | A26-HN | V29-HN | K28-HN | F30-H𝛼 | K28-H𝛼 |
|  | A26-H𝛼 | K28-Hβ2 | K28-Hβ3 | V29-H𝛾1 | V29-Hβ | A26-Hβ |  |  |
|  |  |  |  |  |  |  |  |  |
|  | T25-H𝛾2 | A24-H𝛼/T25-Hβ | F30-Hβ3 |  |  |  |  |  |
| K28E27 | K28-HN | A26-HN | V29-HN | E27-HN | K31-HN | K28-H𝛼 | A26-H𝛼 | E27-H𝛼 |
|  | K28-Hβ3 | V29-Hβ | T25-H𝛾2 | F30-H𝛼 |  |  |  |  |
| F30V29 | K31-HN | F30-HN | V29-HN | Q32-H𝛼 | V29-H𝛼 | A26-H𝛼 | E27-H𝛼 | V29-Hβ |
|  | K31-H𝛾3 | L5-H𝛿/T18-H𝛾2 |  |  |  |  |  |  |
| K31F30 | K31-HN | F30-HN | A34-HN | V29-HN | Y33-H𝛼 | V29-H𝛼 | K28-H𝛼 | V29-Hβ |
|  | V29-H𝛾1 | K31-H𝛾3 |  |  |  |  |  |  |
| Q32K31 | Q32-Hε22 | Q32-H𝛼 | K31-Hβ3 | Y33-Hβ3 | Q32-HN |  |  |  |
| Y33Q32 | K31/D36-HN | Y33/N35-HN | Q32-HN | Q32-Hε2a | Y33-Hh | Y33-H𝛼 | Q32-H𝛼 |  |
|  | N35-Hβ2/3 | Y33-Hβ3 | A34-H𝛼 | V29-H𝛼 |  |  |  |  |
| A34Y33 | D36/A34-HN | Y33/N35-HN | N32-HN | N37-HN | Y33-H𝛿 | Y33-Hε | N35-H𝛼 | Y33-H𝛼 |
|  | Q32-H𝛼 | Y33-Hβ3 | N35-Hβ2/3 | Y33-Hβ3 | N37-Hβ2 | N37-Hβ3 | D36-Hβ2 | A34-H𝛼 |
| N35A34 | A34-HN | D36-HN | N35-HN | V39-HN | G38-HN | N37-HN | N37-H𝛿22 | N35-H𝛼 |
|  | A34-H𝛼 | K31-H𝛼 | N35-Hβ2/3 | A34-Hβ | Q32-H𝛼 |  |  |  |
| D36N35 | N35-HN | D36-HN | N37-HN | N35-H𝛼 | D36-H𝛼 | A34-H𝛼 |  |  |
|  | A34-Hβ | V39-H𝛾2 | N35-Hβ | D36-Hβ3 |  |  |  |  |
| N37D36 | N37-HN | G38-HN | V39-HN | N35-HN | D36-HN | N37-H𝛼 | N35-H𝛼 | D36-H𝛼 |
|  | D36-Hβ2 | D36-Hβ3 | N37-Hβ3 | V39-H𝛾2 | N37-H𝛿22 |  |  |  |
| G38N37 | D36/D40-HN | N37-HN | G38-HN | V39-HN | N37-H𝛿22 | N37-H𝛿21 | N37-H𝛼 | D40-H𝛼 |
|  | G38-H𝛼3 | N37-Hβ2 | N37-Hβ3 | G38-H𝛼2 |  |  |  |  |
| V39G38 | D40-HN | G38-HN | V39-HN | N35-HN | D40-H𝛼 | N37-H𝛼 | G38-H𝛼2 | G38-H𝛼3 |
|  | V39-H𝛾2 | N37-Hβ2 | A34-Hβ |  |  |  |  |  |
| D40V39 | G38-HN | V39-HN | D40-HN | D40-H𝛼 | N35-H𝛼 | V39-H𝛼 | A34-H𝛼 | D40-Hβ2 |
|  | V39-Hβ | A34-Hβ | V39-H𝛾2 | D40-Hβ3 |  |  |  |  |
| G41D40 | D40-HN | G41-HN | E42-HN | D40-H𝛼 | E56-H𝛼 | V39-H𝛼 | G41-H𝛼3 | D40-Hβ2 |
|  | E56-Hβ3 | V39-Hβ | V39-H𝛾1 | D40-Hβ3 |  |  |  |  |
| E42G41 | W43-HN | E42-HN | T55-HN | G41-HN | W43-H𝛿1 | E42-H𝛼 | G41-H𝛼3 | V54-H𝛼 |
|  | T55-Hβ | E42-Hβ2 |  |  |  |  |  |  |
| W43E42 | W43-HN | T44-HN | E42-HN | T55-HN | G41-HN | W43-H𝛿 | G41-H𝛼3 | W43-H𝛼 |
|  | V54-H𝛼 | T55-Hβ | E42-Hβ2 | E42-Hβ3 | E42-H𝛼 |  |  |  |
| T44W43 | W43-HN | T44-HN | Y45-HN | D47-HN | T44-H𝛼 | E42-H𝛼 | W43-H𝛼 | V54-H𝛼 |
|  | W43-Hβ3 | W43-Hβ2 | E42-Hβ2 | T44-Hβ |  |  |  |  |
| Y45T44 | T44-HN | Y45-HN | Y45-Hh | D46-HN | W43-H𝛼 | Y45-H𝛼 | T44-H𝛼 | D46-H𝛼 |
|  | Y45-Hβ3 | T44-H𝛾2 | T44-Hβ | V54-H𝛼 | T53-HN |  |  |  |
| D46Y45 | D46-HN | K50-HN | D47-HN | Y45-HN | T44-HN | T44-Hβ | Y45-Hβ3 | D46-H𝛼 |
|  | D46-Hβ3 | D46-Hβ2 | D47-Hβ2 | D47-H𝛼 |  |  |  |  |
| D47D46 | Y45-HN | D47-HN | D46-HN | T49-HN | K50-HN | T51-HN | A48-HN | D47-H𝛼/T49-Hβ |
|  | D47-Hβ3 | T49/K50-H𝛼 | D46-H𝛼 | D46-Hβ3 | D46-Hβ2 | Y45-H𝛼 | D46-H𝛼 | D47-Hβ2 |
| A48D47 | D46/T51-HN | K50-HN | T49-HN | A48-HN | D47-HN | A48-Hβ | D47-Hβ2 | D47-Hβ3 |
|  | A48-H𝛼 | D46-H𝛼 | T49-H𝛾2 | K50/T49-H𝛼 | K50-Hε |  |  |  |
| T49A48 | T49-HN | D46/T51-HN | K50-HN | A48-HN | D47-HN | A48-Hβ | T49-H𝛾2 |  |
|  | K50-Hε | K50/T49-H𝛼 |  |  |  |  |  |  |
| K50T49 | K50-HN | T49-HN | D46/T51-HN | D47-HN | T51-H𝛼 | A48-Hβ | T49/T51-H𝛾2 | K50-Hε |
|  | K50/T49-H𝛼 | Y3-H𝛼 | K50-Hβ3 |  |  |  |  |  |
| T51K50 | Y45-Hh | K50-HN | T51-Hβ | T50-H𝛼 | Y45-H𝛼 | T51-H𝛼 | T49-H𝛼 |  |
|  | D46/T51-HN | T49-Hβ |  |  |  |  |  |  |
| F52T51 | F52-HN | T53-HN | K50-HN | T51-HN | V54-HN | T49-HN | F52-H𝛼 | T51-H𝛼 |
|  | T51-H𝛾2 | E42-Hβ2 | D46-Hβ3 | F52Hβ2/3 | K50-H𝛼 | T51-Hβ | D46-HN |  |
| T53F52 | T53-HN | F52-HN | F52-H𝛼 | V54-H𝛾 | T51-H𝛾2 |  |  |  |
| V54T53 | T53-HN | T55-HN | V54-HN | T51-HN | E42-HN | V54-H𝛼 | T53-H𝛼 | V54-H𝛾2 |
|  | T55-Hβ | W43-H𝛼 | T53-H𝛾2 | T44-HN |  |  |  |  |
| T55V54 | T53-HN | T44/N8-HN | E42-HN | T55-HN | V54/G9-HN | E56-HN | T55-H𝛾1 | W43-H𝛼 |
|  | V54-H𝛼 | T55-Hβ | V54-H𝛾2 | E42-H𝛼 | N8/T53-H𝛼 |  |  |  |
| E56T55 | E56/G41-HN | V54-HN | T55-HN | E42-HN | N8-HN | T53-HN | E56-H𝛼 | T55-H𝛾2 |
|  | G9-H𝛼3 | E42-H𝛼 | E56-Hβ2 | E42-Hβ2 | V54-H𝛼 | T55-Hβ |  |  |
| Q2εQ2𝛿 | Q2-Hε21 | Q2-Hε22 | Q2-H𝛾2 | Q2-Hβ2 |  |  |  |  |
| N8𝛿N8𝛾 | L7-HN | N8-HN | G9-HN | E56-HN | N8-H𝛿22 | N8-H𝛿21 | N8-H𝛼 | N8-Hβ2 |
|  | N8-Hβ3 |  |  |  |  |  |  |  |
| Q32εQ32𝛿 | Q32-Hε21 | Q32-Hε22 | Q32-H𝛼 | Y33-H𝛼 | Q32-Hβ | K28-Hβ3 | N35-Hβ3 |  |
| N37𝛿N37𝛾 | A34-HN | G38-HN | N37-HN | N37-H𝛿21 | N37-H𝛿22 | N37-H𝛼 | N37-Hβ2 |  |
|  | N37-Hβ3 | A34-Hβ |  |  |  |  |  |  |
| N35𝛿N35𝛾 | N37-H𝛿21 | N35-H𝛼 | N35-Hβ2/3 |  |  |  |  |  |

| Residues  Table S4: Full-list of observed cross-peaks in the (H)NCAH spectrum. * denote overlapping residues | Observed Correlations | | | | | | | | | | | | | | | | | | | |
| --- | --- | --- | --- | --- | --- | --- | --- | --- | --- | --- | --- | --- | --- | --- | --- | --- | --- | --- | --- | --- |
| Q2 |  | 3YHN | | 2QHN | E19H𝛼 | Q2H𝛼 | Q2Hβ2 | | Q2Hβ3 Q2H𝛾2 | | | | |  | |  |  | | |  |
| Y3 |  | Y3Hh | | Y3H𝛼 | Y3Hβ2 | Y3Hβ3 | Y3Hε | |  | | | | |  | |  |  | | |  |
| K4 |  | K4HN | | K4H𝛼 | T17H𝛼 | F52Hβ2/3 | K4Hβ2 | | K4Hβ3 | | | | |  | |  |  | | |  |
| L5 |  | L5Hβ | | L5H𝛾 | L5H𝛿 | I6Hβ | Y3H𝛼 | | L7HN | | | | |  | |  |  | | |  |
| I6 |  | E15H𝛼 | | T53H𝛼 | L5H𝛼 | I6H𝛼 | I6Hβ | | I6H𝛾12 | | I6H𝛾13 | | I6H𝛿 | | | |  | | |  |
| L7 |  | L7H𝛼 | | L7Hβ2/3 | L7H𝛾 | L7H𝛿 | L7HN | | I6H𝛼 | |  | |  | | | |  | | |  |
| N8 |  | N8HN | | N8H𝛼 | N8Hβ2 | N8Hβ3 | G9H𝛼3 | |  | |  | |  | | | |  | | |  |
| G9 |  | G9H𝛼2 | | G9H𝛼3 | G9HN | K13H𝛼 | K10HN | | T55HN | | L12H𝛼 | | L12Hβ2 K10Hβ | | | |  | | |  |
| K10* |  | K10HN | | T11HN | G9HN | K10H𝛼 | G9H𝛼3 | | K10Hβ | | K10H𝛾 | |  | | | |  | | |  |
| T11 |  | T11HN | | T11H𝛼 | T11H𝛾2 | T11Hβ | T11H𝛾1 | |  | |  | |  | | | |  | | |  |
| L12 |  | L7HN | | L12H𝛼 | L12Hβ2 | L12Hβ3 | K13H𝛾 | | L12H𝛿 | |  | |  | | | |  | | |  |
| K13 |  | K13HN | | K13H𝛼 | K13Hε | K13Hβ2 | K13Hβ3 | | K13H𝛿 | | K13H𝛾 | |  | | | |  | | |  |
| G14 |  | G14HN | | K13HN | L7HN | E15H𝛼 | G14H𝛼2 | | G14H𝛼3 | |  | |  | | | |  | | |  |
| E15 |  | T16HN | | L7HN | E15H𝛼 | T16H𝛼 | E15Hβ2 | | E15Hβ3 | |  | |  | | | |  | | |  |
| T16 |  | T16HN | | T17HN | T18HN | T16H𝛼 | T16Hβ | | T16H𝛾2 | | T16H𝛾1 | |  | | | |  | | |  |
| T17 |  | T17HN | | T17H𝛼 | T17Hβ | T17H𝛾2 | T18HN | | T16HN | | T17H𝛾1 | |  | | | |  | | |  |
| T18 |  | E19HN | | T18HN | T17HN | T18H𝛼 | T18Hβ | | T18H𝛾2 | | T18H𝛾1 | | E19H𝛼 | | | |  | | |  |
| E19 |  | E19HN | | E19H𝛼 | E19Hβ2 | E19Hβ3 |  | |  | |  | |  | | | |  | | |  |
| A20 |  | V21HN | | A20HN | A20H𝛼 | A20Hβ |  | |  | |  | |  | | | |  | | |  |
| V21 |  | V21HN | | D22HN | V21H𝛼 | V21Hβ | V21H𝛾1 | | V21H𝛾2 | |  | | | | |  |  | | |  |
| D22 |  | D22H𝛼 | | D22Hβ2 | D22Hβ3 | D22HN | A20HN | | A20Hβ | |  | | | | |  |  | | |  |
| A23 |  | A23H𝛼 | | A23Hβ | A23HN | D22HN | T24HN | |  | |  | | | | |  |  | | |  |
| A24 |  | A24H𝛼 | | A24Hβ | A24HN | T25HN |  | |  | |  | | | | |  |  | | |  |
| T25 |  | T25H𝛼 | | T25Hβ | T25H𝛾2 | T25HN |  | |  | |  | | | | |  |  | | |  |
| A26 |  | A26H𝛼 | | A26Hβ | A26HN | E27HN |  | |  | |  | | | | |  |  | | |  |
| E27 |  | E27H𝛼 | | E27Hβ2 | E27Hβ3 | E27H𝛾 | K28HN | | E27HN | |  | | | | |  |  | | |  |
| K28 |  | K28HN | | K28H𝛼 | K28Hε | K28Hβ2 | K28Hβ3 | | K28H𝛿 | | K28H𝛾 | | | | |  |  | | |  |
| V29 |  | V29HN | | V29H𝛼 | V29Hβ | V29H𝛾1 | V29H𝛾2 | |  | |  | | | | |  |  | | |  |
| F30 | F30HN | | F30H𝛼 | | F30Hβ2 | F30Hβ3 | | F30H𝛿/ε | | V29H𝛾1 | |  | |  |  | | |  | | |
| K31 | K31HN | | K31H𝛼 | | Q32HN | K31Hβ2 | | K31Hβ3 | | K31Hz | | K31H𝛾 | |  |  | | |  | | |
| Q32* | Q32Hε21 | | Q32Hε22 | | Q32H𝛼 | F30HN | | Q32Hβ | |  | |  | |  |  | | |  | | |
| Y33 | Y33HN | | Y33Hβ2 | | Y33Hβ3 | Y33HN | | A34HN | | Y33H𝛼 | | D36HN | | Y33H𝛿 | Y33Hh | | |  | | |
| A34 | A34H𝛼 | | A34Hβ | | A34HN | Y33H𝛼 | | N35H𝛼 | |  | |  | |  |  | | |  | | |
| N35 | N35H𝛼 | | N35Hβ2 | | D35Hβ3 | N35HN | | A34HN | | N37H𝛿21 | |  | |  |  | | |  | | |
| D36 | D36H𝛼 | | D36Hβ2 | | D36Hβ3 | D36HN | | D35H𝛼 | | N37HN | | G38HN | |  |  | | |  | | |
| N37 | N37H𝛼 | | N37Hβ2 | | N37Hβ3 | N37HN | | G38HN | | N37H𝛿21 | | N37H𝛿22 | |  |  | | |  | | |
| G38 | G38H𝛼2 | | G38H𝛼3 | | G38HN | V39HN | | D40HN | | D40H𝛼 | | D36H𝛼 | |  |  | | |  | | |
| V39 | V39H𝛼 | | V39Hβ | | V39H𝛾1 | V39H𝛾2 | | V39HN | | D40HN | | D40H𝛼 | | N35H𝛼 |  | | |  | | |
|  | D40Hβ2 | | D40Hβ3 | | E56H𝛾 |  | |  | |  | |  | |  |  | | |  | | |
| D40 | D40H𝛼 | | D40Hβ2 | | D40Hβ3 | D40HN | | V39H𝛼 | | V39Hβ | |  | |  |  | | |  | | |
| G41 | G41H𝛼2 | | G41H𝛼3 | | G41HN | D40HN | | E42HN | |  | |  | |  |  | | |  | | |
| E42 | E42H𝛼 | | E42Hβ2 | | E42Hβ3 | E42HN | | W43HN | | G41H𝛼2 | |  | |  |  | | |  | | |
| W43 | W43H𝛼 | | W43Hβ2 | | W43Hβ3 | W43HN | | W43Hε1 | | W43Hε3 | | W43H𝛿1 | |  |  | | |  | | |
| T44 | T44H𝛼 | | T44Hβ | | T44H𝛾2 | T44H𝛾1 | | W43HN | | W43H𝛼 | |  | |  |  | | |  | | |
| Y45 | Y45HN | | D46HN | | T44Hβ |  | |  | |  | |  | |  |  | | |  | | |
| D46 | D46H𝛼 | | D46Hβ2 | | D46Hβ3 | D46HN | | D47HN | |  | |  | |  |  | | |  | | |
| D47 | D47H𝛼 | | D47Hβ2 | | D47Hβ3 | D47HN | |  | |  | |  | |  |  | | |  | | |
| A48 | A48H𝛼 | | A48Hβ1 | | A48HN | D47H𝛼 | | D46H𝛼 | |  | |  | |  |  | | | |  | |
| T49 | T49H𝛼 | | T49Hβ | | T49H𝛾2 | T49HN | | A48Hβ | | T51HN | |  | |  |  | | | |  | |
| K50 | K50H𝛼 | | K50HN | | T51HN |  | |  | |  | |  | |  |  | | | |  | |
| T51 | T51H𝛼 | | T51Hβ | | T51H𝛾2 | T51HN | | T53HN | | F52H𝛼 | |  | |  |  | | | |  | |
| F52 | F52H𝛼 | | F52Hβ2/3 | | T53H𝛾2 |  | |  | |  | |  | |  |  | | | |  | |
| T53 | T53H𝛼 | | T53H𝛾2 | | T53HN | V54H𝛼 | | T53Hβ | | 6IHβ | |  | |  |  | | | |  | |
| V54 | V54H𝛼 | | V54H𝛾1 | | V54H𝛾2 | V54Hβ | | V54HN | | T53H𝛼 | | W43H𝛼 | |  |  | | | |  | |
| T55 | T55H𝛼 | | T55Hβ | | T55H𝛾2 | T55HN | | N8Hβ3 | | N8H𝛼 | |  | |  |  | | | |  | |
| E56 | E56H𝛼 | | E56Hβ2 | | E56Hβ3 | E56HN | | E56H𝛾 | |  | |  | |  |  | | | |  | |
